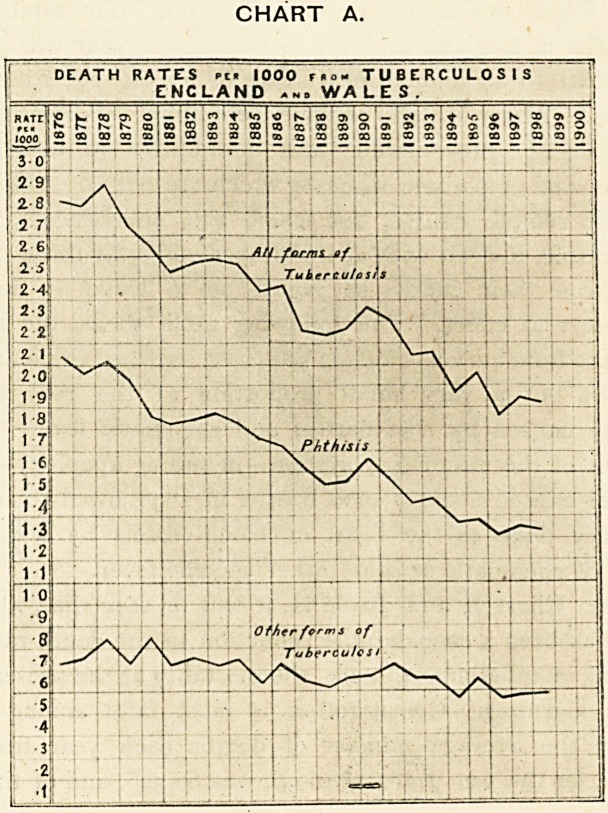# Statistics Concerning Consumption and Other Preventable Diseases

**Published:** 1901-03

**Authors:** W. H. Symons

**Affiliations:** Medical Officer of Health, City of Bath.


### STATISTICS CONCERNING CONSUMPTION AND OTHER PREVENTABLE DISEASES.

BY

W. H. Symons, M.D., D.P.H.,

*Medical Officer of Health, City of Bath.*

---

Now that the crusade against consumption is to be carried on in Gloucester, Somerset, and Wilts, it appears desirable to bring together some figures which may be of service in evoking local interest by comparing the death returns for these three counties with those for England and Wales. The latest returns available are for the year 1898, in the sixty-first Annual Report of the Registrar-General, published in 1900. The total number of deaths in England and Wales during the year was 552,141, equivalent to an annual death-rate of 17.586 per 1,000 living persons. About 164,000 persons were killed by diseases which are obviously preventable; and in addition to these, many persons died of chronic diseases which are not so easily traced to their remote causes, but which are, nevertheless, just as preventable, if dealt with at the proper stage, as those diseases which run their course so rapidly as to leave no doubt as to cause and effect. The following table shows the number of deaths from various causes; but those ascribed to rheumatism must be but a very small proportion of the real number actually caused by rheumatism and rheumatic fever. Many diseases of the circulatory system—including apoplexy—which together caused about 77,000 deaths in 1898, owed their origin to injury done to the heart or blood-vessels by a previous attack of rheumatic or some other specific fever.

From Chart A and Table II. it will be seen that, although there has been a considerable reduction in the death-rate from all forms of tubercular disease, the mortality at the present time is still alarming. Consumption is most fatal among young adults; the average number of deaths each year from this disease, for the ten years 1881–90, was 66,526, and of these no

### TABLE I.

### Deaths in England and Wales, during the year 1898.

| | |
|---|---|
| Deaths from all causes ... | 552,141 |
| ,, ,, Accidents and Violence | 20,278 |
| ,, ,, Preventable Diseases ... | 163,795 |
| ,, ,, Consumption ... | 60,139 |
| ,, ,, Diarrhœa and Enteritis ... | 46,310 |
| ,, ,, Measles ... | 13,220 |
| ,, ,, Influenza ... | 10,405 |
| ,, ,, Whooping Cough ... | 10,175 |
| ,, ,, Diphtheria ... | 7,661 |
| ,, ,, Typhoid or Enteric Fever | 5,708 |
| ,, ,, Scarlet Fever ... | 3,548 |
| ,, ,, Rheumatism ... | 3,320 |
| ,, ,, Puerperal Fever ... | 1,707 |
| ,, ,, Erysipelas ... | 970 |
| ,, ,, Small Pox ... | 253 |
| ,, ,, Minor Preventable Diseases | 469 |

### CHART A.

### TABLE II.

### Death-rates, Tubercular Disease (all forms), Consumption of Lungs, Bowels, and Brain.

MORTALITY AT ALL AGES PER MILLION LIVING.

| | Males. | | Females. | | Reduction per cent. | | Mortality, 1898. | | |
|---|---|---|---|---|---|---|---|---|---|
| | 1861-70 | 1891-97 | 1861-70 | 1891-97 | M. | F. | Males. | Females. | Persons |
| ALL FORMS. | 3,337 | 2,304 | 3,148 | 1,835 | 31 | 42 | 2,175 | 1,671 | 1,916 |
| LUNGS. | 2,467 | 1,598 | 2,483 | 1,259 | 35 | 49 | 1,514 | 1,131 | 1,317 |
| BOWELS. | 320 | 247 | 272 | 208 | 23 | 24 | 220 | 186 | 202 |
| BRAIN. | 412 | 250 | 285 | 196 | 39 | 31 | 237 | 190 | 213 |
| MORTALITY OF CHILDREN UNDER ONE YEAR OF AGE PER MILLION BIRTHS. | | | | | | | | | |
| ALL FORMS. | 10,598 | 9,046 | 8,447 | 7,203 | −15 | −15 | 8,428 | 6,845 | 7,648 |
| LUNGS. | 1,437 | 647 | 1,299 | 550 | −55 | −58 | 572 | 495 | 534 |
| BOWELS. | 4,112 | 4,278 | 3,474 | 3,454 | + 4 | − 1 | 3,834 | 3,229 | 3,536 |
| BRAIN. | 4,239 | 2,446 | 2,974 | 1,795 | −42 | −40 | 2,409 | 1,832 | 2,150 |
| | Males. | | Females. | | Difference '61-70 to '91-97. | | Males. | Females. | Persons |

fewer than 43,832 deaths were those of persons between the ages of 15 and 65 years, the wage-earning period of life.

The question of disease-prevention is very largely a financial question; and when a sufficient number of persons can be made to see it pays to prevent disease the financial difficulty will disappear. Sanitary reformers have received most encouragement and made most progress in preventing the acute diseases of adults. Expense is scarcely considered in fighting plague, cholera, small pox, or epidemic typhoid fever; but children are allowed to die by tens of thousands every year from consumption, diarrhœa, measles, and whooping cough, without any great effort being made to save them. Such an unnecessary loss of life will not be tolerated when the nation wakes up to the importance of saving infant life, in order to counteract the diminution of the birth-rate and to keep up the position of England as a Great Power. From 1881–90 average of 66,526 deaths every year from consumption, the number of deaths has been reduced to 60,139 in the year 1898; namely, 41,335 deaths from phthisis or consumption of the lungs, and 18,804 deaths from other forms of tuberculosis. Compared with these figures, the loss of life in military operations is very small. *The Times*

on January 5th, 1901, reprinted the return of deaths, the total number of men killed in action or dying in the service of the army during seven years' war with France, 1793 to 1800; and the total number of deaths was 48,971, or considerably less than the number of persons killed in one year, year after year, by consumption. The war in South Africa has been a bloody war, but the total reported as killed in action, died of wounds or disease, up to the end of December was 12,158; and of these, 7,185 died of disease, mostly preventable. Consumption kills nearly five times as many every year. The following tables will show the numbers killed in the three counties, Gloucester, Somerset, and Wilts, and in the registration districts of Somerset, and the percentage of deaths from tuberculosis to deaths from all causes.

The number of actual deaths are given for the year 1898, and the mean annual number of deaths and mean death-rates for the ten years 1881–90. The death-rates for deaths from all causes are for standard mean population—the mortality being calculated for each age group separately, and then for a population of 1,000 having the same proportion of each age group as the population of England and Wales. The variations caused in the crude death-rate by different proportions of young and old people are thus eliminated. The Registrar-General does not publish corrected death-rates for special diseases, and the death-rates from tuberculosis for different districts are not strictly comparable without this correction for age grouping. Table V. shows the average number of deaths in the three counties at various age periods, and we find that between the ages of 15 and 35 years, the most enjoyable period of life, consumption causes nearly one-half the total number of deaths.

Until recently the Registrar-General did not make any correction for persons dying in the hospitals at Bath, but belonging to other districts, having only been brought into Bath for treatment, so the death returns for the city appear slightly higher than they really are. The following will show the corrections for 1898:—

| | |
|---|---|
| Crude or uncorrected death-rate per 1,000 persons | 16.3 |
| Death-rate per 1,000 standard population ... | 15.3 |

*TABLE III.*

### Mortality from All Causes and from Tuberculosis.

MEAN ANNUAL DEATH-RATES FROM ALL CAUSES, 1881-90, PER 1,000, STANDARD POPULATION.
MEAN ANNUAL CRUDE DEATH-RATES FOR ALL FORMS OF TUBERCULOSIS, 1881-90, PER 1,000 OF EACH SEX, AND OF PERSONS.
MEAN ANNUAL NUMBER OF DEATHS FOR 1881-90, AND PERCENTAGE OF DEATHS FROM TUBERCULOSIS.
NUMBER OF DEATHS FROM ALL CAUSES AND FROM TUBERCULOSIS IN THE YEAR 1898.

| District and Area in Acres. | Sex. | Population—Census 1891. | Mean Death-Rates, 1881-90. | | Mean Number of Deaths Yearly, 1881-90. | | Tuberculosis. | Deaths, 1898. | |
|---|---|---|---|---|---|---|---|---|---|
| | | | All Causes. | Tuber. | All Causes. | Tuber. | per cent. | All Causes. | Tubercular. |
| ENGLAND— | M. | 14,052,901 | 20.22 | 2.62 | 269,832 | 35,000 | 13 | 283,981 | 33,098 |
| 37,317,885 | F. | 14,949,624 | 18.01 | 2.23 | 254,646 | 31,527 | 12.4 | 268,160 | 27,041 |
| Acres. | P. | 29,002,525 | 19.08 | 2.42 | 524,478 | 66,526 | 12.7 | 552,141 | 60,139 |
| THREE COUNTIES | M. | 619,675 | 16.93 | 2.06 | 11,268 | 1,247 | 11.1 | 10,552 | 1,063 |
| 2,592,156 | F. | 694,466 | 15.06 | 1.83 | 11,240 | 1,243 | 11.1 | 10,538 | 934 |
| Acres. | P. | 1,314,081 | 16.08 | 1.93 | 22,508 | 2,489 | 11.1 | 21,090 | 1,997 |
| GLOUCESTER— | M. | 255,341 | 18.09 | 2.16 | 4,781 | 542 | 11.3 | 4,585 | 504 |
| 709,622 | F. | 293,545 | 15.75 | 1.91 | 4,787 | 546 | 11.4 | 4,531 | 410 |
| Acres. | P. | 548,886 | 16.89 | 2.02 | 9,568 | 1,088 | 11.4 | 9,116 | 914 |
| WILTS— | M. | 125,696 | 15.14 | 1.95 | 2,137 | 242 | 11.3 | 1,957 | 180 |
| 809,220 | F. | 129,423 | 14.76 | 1.89 | 2,126 | 242 | 11.4 | 1,870 | 167 |
| Acres. | P. | 255,119 | 14.95 | 1.92 | 4,262 | 484 | 11.4 | 3,807 | 347 |
| SOMERSET— | M. | 238,638 | 16.61 | 1.97 | 4,350 | 463 | 10.6 | 4,030 | 379 |
| 1,073,314 | F. | 271,438 | 14.46 | 1.71 | 4,328 | 454 | 10.5 | 4,137 | 357 |
| Acres. | P. | 510,076 | 15.50 | 1.83 | 8,678 | 917 | 10.6 | 8,167 | 736 |

### TABLE IV.

### Registration Districts of Somerset.

MEAN ANNUAL DEATH-RATES FROM ALL CAUSES AND FROM TUBERCULOSIS, 1881–90, PER 1,000 PERSONS LIVING, AT ALL AGES.

MEAN ANNUAL DEATHS, 1881–90, WITH PERCENTAGE OF DEATHS FROM TUBERCULOSIS.

DEATHS FROM ALL CAUSES AND FROM TUBERCULOSIS IN THE YEAR 1898.

| District. | Area in Acres. | Population, 1891. | Mean Death-Rates, 1881-90. | | Mean Deaths, 1881-90. | | | Deaths, 1898. | |
|---|---|---|---|---|---|---|---|---|---|
| | | | | | All Causes | Tuberculosis. | | | |
| | | | All Causes. | Tuber. | Deaths. | Deaths. | Per Cent. | All Causes. | Tuber. |
| WILLITON ... ... ... ... | 100,679 | 17,155 | 13.38 | 1.43 | 292 | 26 | 8.8 | 237 | 24 |
| DULVERTON ... ... ... ... | 78,980 | 4,922 | 12.67 | 1.43 | 80 | 8 | 9.3 | 65 | 9 |
| WELLINGTON ... ... ... | 61,093 | 18,410 | 14.31 | 1.72 | 326 | 32 | 9.9 | 289 | 26 |
| TAUNTON ... ... ... ... | 73,109 | 36,779 | 16.75 | 1.89 | 680 | 69 | 10.0 | 643 | 67 |
| BRIDGWATER ... ... ... | 88,475 | 32,758 | 14.31 | 1.75 | 561 | 59 | 10.5 | 522 | 33 |
| LANGPORT ... ... ... ... | 59,410 | 14,478 | 14.47 | 1.68 | 264 | 25 | 9.6 | 210 | 17 |
| CHARD ... ... ... ... ... | 57,451 | 24,825 | 15.48 | 1.96 | 435 | 49 | 11.3 | 341 | 35 |
| YEOVIL ... .. ... ... ... | 55,752 | 28,431 | 15 23 | 1.80 | 469 | 50 | 10.6 | 372 | 30 |
| WINCANTON ... ... ... ... | 64,540 | 18,251 | 14 80 | 1.76 | 337 | 34 | 10.2 | 214 | 16 |
| FROME ... ... ... ... ... | 52,752 | 22,850 | 14.61 | 1.45 | 389 | 33 | 8.6 | 371 | 32 |
| SHEPTON MALLET ... ... | 50,109 | 15,559 | 14.51 | 1.44 | 268 | 23 | 8.4 | 197 | 11 |
| WELLS ... ... ... ... ... | 66,646 | 23,856 | 16.82 | 2.00 | 441 | 46 | 10.3 | 450 | 38 |
| AXBRIDGE ... ... ... ... | 97,436 | 43,704 | 14.56 | 1.99 | 693 | 83 | 12.0 | 742 | 71 |
| CLUTTON ... ... ... ... | 47,897 | 24,904 | 14.43 | 1.41 | 412 | 34 | 8.3 | 373 | 35 |
| BATH ... ... ... ... ... | 30,727 | 75,196 | 17.14 | 1.95 | 1,350 | 143 | 10.6 | 1,238 | 115 |
| KEYNSHAM ... ... ... ... | 33,570 | 29,885 | 15.58 | 2.16 | 466 | 60 | 12.9 | 534 | 48 |
| BEDMINSTER ... ... ... ... | 54,688 | 77,574 | 16.45 | 1.98 | 1,214 | 144 | 11.8 | 1,369 | 129 |

### TABLE V.

### Mortality from All Causes and from Tuberculosis.

AVERAGE ANNUAL NUMBER OF DEATHS FOR 1881-90, AT VARIOUS AGES.
PERCENTAGE OF DEATHS FROM TUBERCULOSIS TO DEATHS FROM ALL CAUSES.

| | | All Ages. | Under 5. | 5-15. | 15-25. | 25-35. | 35-45. | 45-65. | 65 & over. |
|---|---|---|---|---|---|---|---|---|---|
| ENGLAND AND WALES— Mean Population, 27,488,482 | All Causes. | 524,477 | 200,998 | 26,401 | 25,906 | 30,755 | 35,784 | 89,839 | 114,795 |
| | Tuberculosis. | 66,526 | 15,914 | 5,251 | 11,082 | 12,375 | 10,153 | 10,222 | 1,530 |
| | Percentage. | 12.7 | 7.9 | 19.9 | 42.8 | 40.2 | 28.4 | 11.3 | 1.3 |
| THREE COUNTIES— Mean Population, 1,289,305 | All Causes. | 22,508 | 6,734 | 1,026 | 1,061 | 1,168 | 1,363 | 3,989 | 7,167 |
| | Tuberculosis. | 2,489 | 476 | 211 | 473 | 493 | 373 | 392 | 71 |
| | Percentage. | 11.1 | 7.1 | 20.6 | 44.6 | 42.2 | 27.4 | 9.8 | 1.0 |
| GLOUCESTER— Mean Population, 537,026 | All Causes. | 9,568 | 3,100 | 453 | 450 | 512 | 608 | 1,677 | 2,769 |
| | Tuberculosis. | 1,088 | 220 | 97 | 196 | 210 | 167 | 170 | 29 |
| | Percentage. | 11.4 | 7.0 | 21.4 | 43.6 | 41.0 | 27.5 | 10.1 | 1.0 |
| WILTS— Mean Population, 215,940 | All Causes. | 4,262 | 1,157 | 187 | 199 | 221 | 242 | 774 | 1,482 |
| | Tuberculosis. | 484 | 93 | 39 | 91 | 97 | 68 | 80 | 16 |
| | Percentage. | 11.4 | 8.0 | 20.9 | 45.7 | 43.9 | 28.1 | 10.3 | 1.0 |
| SOMERSET— Mean Population, 500,339 | All Causes. | 8,678 | 2,477 | 387 | 412 | 435 | 513 | 1,538 | 2,916 |
| | Tuberculosis. | 917 | 162 | 75 | 187 | 187 | 137 | 142 | 26 |
| | Percentage. | 10.6 | 6.5 | 19.4 | 45.4 | 43.0 | 26.7 | 9.2 | 0.9 |
| BATH DISTRICT— Mean Population, 72,970 | All Causes. | 1,350 | 341 | 51 | 61 | 72 | 96 | 266 | 463 |
| | Tuberculosis. | 143 | 22 | 11 | 25 | 28 | 27 | 28 | 3 |
| | Percentage. | 10.6 | 6.5 | 21.6 | 41.0 | 38.9 | 28.1 | 10.5 | 0.7 |

| | |
|---|---|
| Death-rate corrected for persons dying in hospitals ... ... ... ... ... ... ... ... | 14.1 |
| Death-rate from phthisis per 1,000 persons ... | 1.12 |
| Death-rate from phthisis per 1,000 standard population ... ... ... ... ... ... ... | 1.06 |
| Death-rate from other tubercular diseases per 1,000 persons ... ... ... ... ... ... ... ... | 0.46 |
| Death-rate from other tubercular diseases per 1,000 standard population ... ... ... | 0.62 |

The occupations followed by the population have considerable influence on the death-rate from consumption in any locality; but it is not easy to determine how far the disease is the result of the occupation. Weakly persons will naturally choose indoor occupations, unless they are better advised; while the robust will be inclined to out-of-door work, and will be less likely to suffer from consumption than those who get impure air. Table VI. gives the percentages of deaths from phthisis to deaths from all causes at various age periods among different classes for the years 1890-2. These are calculated from figures given in Part II. of the Supplement to the *Fifty-fifth Annual Report of the Registrar-General*, p. 150:—

TABLE VI.

**Comparative Mortality from Phthisis in various Occupations, 1890-92.**

NUMBER OF DEATHS FROM PHTHISIS PER HUNDRED DEATHS FROM ALL CAUSES.

| Age Period:— | 15 to 20 | 20 to 25 | 25 to 35 | 35 to 45 |
|---|---|---|---|---|
| All Males ... ... .. | 28 | 39 | 36 | 27 |
| Miners ... ... ... ... | 16 | 25 | 20 | 16 |
| Merchant Seamen ... | 17 | 26 | 29 | 20 |
| Farmers ... ... ... ... | 31 | 35 | 29 | 22 |
| Farm Labourers ... ... | 24 | 36 | 35 | 26 |
| Builders ... ... ... ... | 30 | 38 | 38 | 32 |
| Shopkeepers ... ... ... | 28 | 48 | 40 | 29 |
| Commercial Clerks ... | 40 | 52 | 45 | 32 |
| School Teachers ... ... | 54 | 52 | 41 | 31 |

The geographical distribution of tubercular disease shows great variety, as may be seen by comparing the mean annual death-rates from phthisis for registration counties, the death-rates being properly corrected for the age and sex distribution of the populations of the counties. During 1881-90 the average annual death-rate from pulmonary phthisis in England and Wales was 1.7; during the same period the rate for North Wales was 2.1, and for the county of Worcester 1.2 per 1,000. Taking the death-rate in England from phthisis as 1,000, the comparative mortality in North Wales was 1,255, while in Worcester the comparative mortality from phthisis was 706. All manner of explanations, based chiefly upon geological and meteorological considerations, have been offered to account for these variations. Dr. Alfred Haviland ascribed the low death-rate from consumption, among populations living in sheltered valleys, to protection from strong winds. Dr. Haviland suggests a fatal element in these winds: "such as *ozone*, which finds its way straight to the tuberculous matter, attacks it, inflames its surroundings, and sloughs it out, killing the host, just like the lymph of Dr. Koch, which, when injected, is said instinctively to rush off to seize its quarry, *tubercle*."[1] A more modern explanation of the variation is that persons living in places exposed to the force of the wind keep the doors and windows of their houses shut, and so really get less fresh air, less ozone, than those who live in more sheltered spots, where the doors and windows may be freely opened. Other observers consider the chief local predisposing cause of phthisis to be dampness of subsoil and flatness of the ground. Dr. Buchanan showed that great diminution in the number of deaths from phthisis followed the laying of main drains in various towns; for example, he quoted the reduction in the death-rates by phthisis in the following towns among others:—Salisbury, 49 per cent. of its previous rate; Cheltenham, 26; and Bristol, 22 per cent. These figures help to prove that natural defects in subsoil may be overcome by artificial means. Occupation and education, the capability of providing large rooms, good food and attention for children and

[1] *The Geographical Distribution of Disease in Great Britain*, 2nd Ed., 1892, p. 29.

old persons have probably far more influence on the death-rate than any circumstance connected with the configuration of the ground in most English towns. The death-rate may be a measure of the average sanitary condition of a town or district; but it is of very little importance to an individual or family going to reside in the district, the condition of the particular house chosen as a dwelling is far more important.

Groups of houses in any districts will be found to differ, as regards prevalence of phthisis, far more than any two districts; and if the incidence of tubercular diseases among the inhabitants of any street of badly constructed houses is noted for a sufficient length of time, and spot-maps of deaths from tuberculosis prepared, the distribution of the deaths will be found to be in groups, somewhat similar to the grouping of infectious cases, and different to the arrangement noticed when deaths from non-infectious endemic diseases are studied. The number of multiple deaths occurring in houses will be largely in excess of the number calculated by any of the mathematical formulæ commonly used for estimating the probable recurrence of any event. Thus, in a period of 32 years, there were among 9,000 occupied houses in Bath 1,813 houses in which one death from some tubercular disease occurred, and there were 473 houses with two such deaths, the probable number being calcuated as 108, or less than one-fourth the observed number. There were 112 houses with three deaths, the calculated number being six, and 47 houses with four deaths from tuberculosis, while, by chance, there should have been no house with four deaths from this cause during 32 years.

The above statistics only show the number of deaths. Many persons suffer from tubercular disease who do not die from this cause, and there must be at least 6,000 persons in the counties of Gloucester, Somerset, and Wilts who are suffering from consumption at the present time. What can we do to diminish this suffering and fearful loss of life? The notification of cases of consumption, the provision of sanatoria, disinfection and improvement of rooms occupied by the consumptive, surgical attention to the throats of children, and the avoidence of dust in occupied rooms are required in addition to ordinary precautions.

The infectiousness of consumption is practically limited to the dried sputum of the consumptive person—the matter expectorated from the lungs,—if this is rendered harmless, there is no danger to others, and the chance of re-infection of the consumptive is reduced. The breath of a consumptive person is not infectious, and there is no good reason why such a person should be shut off from society or from work, if proper precautions are taken; but a careless or ignorant consumptive person is a source of great danger.

A sanatorium for consumptives is certainly not injurious to the surrounding population, its influence is altogether in the other direction. Sanatoria are built far away from great towns to secure a pure atmosphere and to protect the occupants from chance infection of common catarrhs and influenza. The isolation of the consumptive for a few months is recommended for the benefit of the patient, not for the protection of the public.

The general public can be best protected by securing healthy sanitary conditions as to food and food stores; by cleanliness of person, house, and town; and, above all, by recognising fresh air as the most important of all foods. All windows should be made to open freely, especially the upper sashes; if these are fixed, the upper part of the room cannot be properly ventilated or swept out by fresh air. We carefully protect our drinking water from pollution with sewage; it is quite as important to keep the atmosphere we breathe free from the sewage or waste products of the lungs. Persons living in an impure atmosphere are easily infected by many diseases, and life is not only shortened, but rendered unpleasant. Our endeavour should be directed to increasing the resisting power of the individual, so that he may withstand infectious matter just as the rocks withstand the ocean waves.

## Figures and Tables

**CHART A. f1:**